# Pulmonary tumor thromboembolism: A case report and review of literature

**DOI:** 10.4103/1817-1737.36553

**Published:** 2007

**Authors:** Vinod Aiyappan, A. Alwail

**Affiliations:** Specialist Registrar in Respiratory Medicine, Queen Elizabeth Hospital NHS Trust, Kings Lynn, UK; * *Bassetlaw Hospital, Worksop, Nottinghamshire, UK*

**Keywords:** Occult carcinoma, tumor thromboembolism

## Abstract

Pulmonary tumor thromoembolism is well described in literature especially in patients known to have cancer. We are presenting this report of a patient presenting with Acute Cor-pulmonale secondary to Occult Gastric carcinoma causing pulmonary tumor embolism. This is an unusual presentation of occult neoplasm.

Pulmonary tumor embolism is an important but ‘uncommon’ cause of dyspnea in patients with cancer. It is ‘uncommon’ as it is under-recognized! Autopsy reports show that up to 26% of patients who die with cancer have tumor cells in their pulmonary vasculature. By this article we aim to increase the awareness of this condition, thereby leading to appropriate management.

## Case Report

A 77-year-old Caucasian male who was admitted in the surgical ward with complaints of abdominal pain and altered bowel habits of 3-week duration was referred to the medical team due to increasing dyspnea. He had sustained an injury to knee 4 weeks back and had been taking regular codeine and paracetamol tablets for pain. His abdomen X-ray had shown fecal loading, rigid sigmoidoscopy was normal and the provisional diagnosis was constipation secondary to codeine. He gave a history of gradually progressive dyspnea over 2 weeks. There was no other significant past medical history. On examination he had features of right-sided heart failure with pulmonary hypertension. Arterial blood gases showed hypoxia. ECG showed S1Q3T3 pattern. The diagnosis of pulmonary embolism was made and he was started on full-dose low- ‘molecular weight’ Heparin. Echocardiogram showed dilated right ventricle with impaired function and right ventricular systolic pressure >80 mmHg. CT pulmonary angiogram showed no evidence of pulmonary embolism but did show multiple lymph node enlargement. Patient had cardiac arrest and resuscitation attempts were unsuccessful.

Postmortem showed multiple lymph node enlargements inside mediastinum and abdomen, malignant-looking ulcer in stomach and tumor infiltration throughout small bowel mesentery. Microscopy of gastric ulcer revealed adenocarcinoma.

Right ventricle did not show any features of hypertrophy.

Macroscopically, lungs were edematous but pulmonary arteries showed no evidence of pulmonary embolism. Microscopy of pulmonary parenchyma showed pulmonary hypertensive changes and extensive tumor thromboemboli involving pulmonary microvasculature.

Final diagnosis: Widespread carcinamatosis due to carcinoma stomach.

Acute cor pulmonale due to pulmonary tumor thromboembolism.

## Discussion

The entity of pulmonary tumor thromboembolism has been described as early as in 1897 (Schmidt).[1] The two routes for pulmonary tumor embolization are through abdominal vessels to the inferior vena cava and through the thoracic duct to the superior vena cava.[2]

The most common presenting complaint is dyspnea.[1] Other features of pulmonary hypertension and cor pulmonale are usually present. Gastrointestinal symptoms precede respiratory symptoms by a median of 2 weeks.[2] ABG shows hypoxemia. ECG shows features of right heart strain and echocardiography shows features of pulmonary hypertension. Symptoms tend to be much severe than physical findings.

The incidence of pulmonary tumor thromboembolism is between 3 and 26% in patients with cancer,[1] but there have been only seven cases of occult gastric cancer presenting as cor pulmonale reported in English literature.

The pulmonary arteries are grossly normal but microscopy reveals fibrocellular intimal proliferation with smooth muscle colonization of luminal neoplastic lesions and associated microthrombi.[3] This causes narrowing of pulmonary arterioles and results in pulmonary hypertension.

Hypoxemia with normal chest radiograph is the common finding. Ventilation perfusion scanning characteristically shows multiple subsegmental mismatched defects. CT pulmonary angiography, which is emerging as the investigation of choice in pulmonary embolism, is not a helpful test in this case. Pulmonary angiography, which is the gold standard to diagnose pulmonary embolism, has poor sensitivity and specificity.[1] Right heart catheterization and pulmonary artery cytology can confirm the diagnosis.[1]

The median survival of these patients reported in literature is 3 days.[2] Thrombolytic therapy is not helpful as pulmonary hypertension is predominantly due to fibrocellular intimal proliferation.[4] Antemortem diagnosis is extremely difficult and there is no effective treatment for this condition. The diagnosis will help in directing the treatment towards effective palliation.
